# Use of proxy indicators for automated surveillance of severe acute respiratory infection, the Netherlands, 2017 to 2023: a proof-of-concept study

**DOI:** 10.2807/1560-7917.ES.2024.29.27.2300657

**Published:** 2024-07-04

**Authors:** Maaike C Swets, Annabel Niessen, Emilie P Buddingh, Ann CTM Vossen, Karin Ellen Veldkamp, Irene K Veldhuijzen, Mark GJ de Boer, Geert H Groeneveld

**Affiliations:** 1 Department of Infectious Diseases, Leiden University Medical Center, Leiden University, Leiden, the Netherlands; 2 National Institute for Public Health and Environment (RIVM), Bilthoven, the Netherlands; 3 Department of Pediatrics, Willem-Alexander Children's Hospital, Leiden University Medical Center, Leiden, the Netherlands; 4 Department of Medical Microbiology, Leiden University Medical Center, Leiden, the Netherlands; 5 Department of Clinical Epidemiology, Leiden University Medical Center, Leiden, the Netherlands; 6 Department of Internal Medicine- Acute Internal Medicine, Leiden University Medical Center, Leiden, the Netherlands

**Keywords:** severe acute respiratory infection (SARI) surveillance, pandemic preparedness, routine data, hospital capacity, contact and droplet precautions

## Abstract

**Background:**

Effective pandemic preparedness requires robust severe acute respiratory infection (SARI) surveillance. However, identifying SARI patients based on symptoms is time-consuming. Using the number of reverse transcription (RT)-PCR tests or contact and droplet precaution labels as a proxy for SARI could accurately reflect the epidemiology of patients presenting with SARI.

**Aim:**

We aimed to compare the number of RT-PCR tests, contact and droplet precaution labels and SARI-related International Classification of Disease (ICD)-10 codes and evaluate their use as surveillance indicators.

**Methods:**

Patients from all age groups hospitalised at Leiden University Medical Center between 1 January 2017 up to and including 30 April 2023 were eligible for inclusion. We used a clinical data collection tool to extract data from electronic medical records. For each surveillance indicator, we plotted the absolute count for each week, the incidence proportion per week and the correlation between the three surveillance indicators.

**Results:**

We included 117,404 hospital admissions. The three surveillance indicators generally followed a similar pattern before and during the COVID-19 pandemic. The correlation was highest between contact and droplet precaution labels and ICD-10 diagnostic codes (Pearson correlation coefficient: 0.84). There was a strong increase in the number of RT-PCR tests after the start of the COVID-19 pandemic.

**Discussion:**

All three surveillance indicators have advantages and disadvantages. ICD-10 diagnostic codes are suitable but are subject to reporting delays. Contact and droplet precaution labels are a feasible option for automated SARI surveillance, since these reflect trends in SARI incidence and may be available real-time.

**Key public health messages**

**What did you want to address in this study and why?**

Surveillance of severe acute respiratory infections (SARI) is important for disease control and prevention. Traditionally, specific diagnostic codes (ICD-10 codes) are used to monitor the number of patients with SARI in hospitals. We wanted to explore other ways to monitor this number. Contact and droplet precaution labels are used to isolate hospitalised patients with a suspected respiratory viral infection, and could be an alternative for SARI surveillance.

**What have we learnt from this study?**

We compared three surveillance indicators (ICD-10 codes, contact and droplet precaution labels and the number of PCR tests) for over 100,000 patients at one hospital in the Netherlands and found that they generally followed a similar pattern. Contact and droplet precaution labels reflect the epidemiology of hospitalised patients presenting with severe viral respiratory disease.

**What are the implications of your findings for public health?**

Using contact and droplet precautions labels from electronic medical records is a feasible option for SARI surveillance. Since these labels can be automatically collected and are available real-time, this could reduce the burden on staff to collect data, increase the speed at which data can be shared between institutions and allow for robust, automated syndromic SARI surveillance.

## Introduction

Severe acute respiratory infection (SARI) surveillance is essential for disease control and prevention, enabling assessment of the effectiveness of community-based preventive measures, detection of unusual events, identification of risk factors and evaluating pandemic preparedness and capacity management [1,2]. Ideally, a SARI surveillance system should be (near) real-time, combining syndromic surveillance with pathogen testing and be automated where possible to decrease the administrative burden. European-level SARI surveillance is available, with weekly reports being published online by the European Centre for Disease Prevention and Control (ECDC) [3]. However, the number of contributing countries is small, and there is often a delay in reporting because of the intensive nature of data collection. At present, there is no robust sentinel or universal SARI surveillance system in the Netherlands.

The rapid developments in the field of data science and the increase in easily accessible healthcare data bring new opportunities for infectious disease surveillance [4]. While manual reporting of cases was once the sole method for infectious disease surveillance, a variety of data sources is used at present [4]. Selected International Classification of Disease (ICD)-10 codes are used in multiple European countries for SARI surveillance, with or without virological test results [5-8]. Although ICD-10 codes are standardised, delays in reporting and the mix of codes being used may over- or underestimate the true number of SARI cases [1]. A narrow selection of codes could underestimate the true number of SARI cases, while a broad selection of codes could overestimate the true number of SARI cases.

Early in the COVID-19 pandemic in 2020, the World Health Organization (WHO) issued guidelines for the protection of healthcare workers, e.g. in hospitals [9]. These guidelines recommended contact and droplet precautions when caring for suspected COVID-19 patients. These guidelines have been implemented rapidly and, in most hospitals in the Netherlands, these patients have a contact and droplet precaution label in their electronic medical record (EMR). During the pandemic, information about the numbers in contact and droplet isolation have been used to determine the COVID-19 impact on hospital capacity [10]. Both before and following the pandemic, contact and droplet isolation precautions have been used for patients with a suspected viral respiratory infection. The number of these patients is likely to reflect the number of patients who are hospitalised with a respiratory tract infection and could serve as a proxy in SARI surveillance. In addition, patients who are hospitalised with a suspected viral respiratory infection are typically tested using a reverse transcription (RT) PCR test. The number of RT-PCR tests for viral respiratory pathogens, irrespective of the test result, could also reflect the number of hospitalised patients with a respiratory tract infection, and be suitable for SARI surveillance.

In this proof-of concept study, we hypothesise that both the number of RT-PCR tests and contact and droplet precaution labels are indicative of SARI and could be pragmatic indicators for monitoring of trends and capacity management in SARI surveillance. Using data between 2017 and 2023 from one hospital in the Netherlands, we compare RT-PCR, contact and droplet precaution labels, and ICD-10 codes to assess SARI counts and incidence proportions and to evaluate their suitability as surveillance tool.

## Methods

### Study design and population

We conducted a retrospective observational study at the Leiden University Medical Center (LUMC, Leiden, the Netherlands), a tertiary university hospital in one of the larger metropolitan areas of the Netherlands. Almost 21,000 patients are admitted to the LUMC every year. Patients of all ages hospitalised for at least 24 h between 1 January 2017 and 30 April 2023 were included.

Patients who were hospitalised for less than 24 h at the LUMC but were transferred to another hospital were included in our study. A patient could be included multiple times, if more than one hospitalisation occurred within the study period, with the exception of readmissions within 10 days of the previous hospitalisation. For all included hospitalisations, we collected data on the presence of the three different surveillance indicators detailed below. 

The study period was divided into three timeframes. The first period (pre-COVID-19) consists of data from week 1 2017 to week 8 2020. The second period starts in week 9 2020, when the first COVID-19 case was reported in the Netherlands and includes data up to week 53 2020. As the registration policy for contact and droplet precaution labels was changed at the end of 2020 (see below), we included a third time period, in which these changes were fully implemented. The third period starts in week 1 2021 and ends at the end of our study period (week 18 2023).

### Surveillance indicators

#### ICD-10 diagnostic codes

ICD-10 diagnostic codes [8] indicative of conditions seen in SARI patients were selected. These codes included: J00–J22 (upper and lower respiratory tract infections), U07.1 and U07.2 (COVID-19 infections). For children, J40 (bronchitis), J45.9 (asthma, unspecified) and J98.8 (other respiratory disorders) are frequently used for SARI in our hospital and were therefore included. In order to be included, ICD-10 codes had to be registered between hospital admission and 7 days after hospital discharge and be registered by the treating physician in the EMR. To avoid the inclusion of patients with chronic disease, mainly asthma, we only included ICD-10 codes if the same ICD-10 code was not registered in the previous year.

#### RT-PCR testing

Patients were tested for respiratory viruses at the discretion of the treating physician. RT-PCR tests were conducted on upper respiratory tract samples, using either a nasal, nasopharyngeal, or throat swabs. Virology results were recorded in the Global Laboratory Information Management System (GLIMS), which is linked to the EMR. The total number of RT-PCR tests performed for one of the following respiratory viruses were collected: human adenovirus, bocavirus, human coronaviruses (SARS-CoV-2, MERS, 229E, HKU1, NL63, OC43), human metapneumovirus, influenza viruses A and B, parainfluenza virus (PIV) 1–4, human rhinovirus and respiratory syncytial virus (RSV). Even though patients were frequently screened for multiple viruses using the multiplex RT-PCR method, we accounted for it as a single RT-PCR test per patient in our analysis. If a patient had more than one (multiplex) RT-PCR test done during hospitalisation, we selected the first test. If a patient tested positive for multiple pathogens within a single RT-PCR test, we included all of the identified pathogens in the virological test results. Only RT-PCR tests that were done 48 h before admission to 48 h after admission were eligible for inclusion to minimise the probability of including hospital-acquired infections. Tests before hospital admission were included to account for patients that were tested, e.g. at the emergency department or in the outpatient clinic, but initially sent home before being readmitted within the next 2 days because of clinical deterioration. RT-PCR test results were reported as positive or negative for each tested virus.

#### Contact and droplet precautions

According to the standard procedure in our hospital, contact and droplet precautions are applied for all patients suspected or confirmed to have a respiratory viral infection from one of the viruses mentioned above. However, for rhinoviruses, these precautions are only recommended for immunocompromised patients and neonates.

The installation of contact and droplet precautions are recorded in the EMR. The process for recording contact and droplet precautions in our hospital’s system underwent a revision on 1 December 2020. Prior to this date, only the infection control and hospital hygiene department staff could add these precautions to the EMR. Precautions were added to the EMR only after a positive RT-PCR test result. Starting from 1 December 2020, a broader range of healthcare personnel, including nurses and physicians from all departments, could add contact and droplet precautions to the EMR. Precautions were taken and added to the EMR for both suspected or confirmed infections. Only contact and droplet precautions registered within 48 h of hospital admission were counted, in order to minimise the probability of including hospital-acquired infections.

### Data collection

CTcue (IQVIA) is a clinical data collection tool that can be used to identify patients and extract data from their EMRs. We collected (structured) data for the following variables: age, date of hospital admission and date of hospital discharge, ICU admission during hospitalisation and information on our three surveillance indicators (ICD-10, RT-PCR and contact and droplet precautions), as described above. The clinical data collection tool was previously validated using Dutch EMR data and showed high accuracy [11,12]. In order to validate the accuracy of our data collection, we selected 2 random weeks for each surveillance indicator, and checked the results with regular quality control data in our hospital. This was done to ensure that the data collection tool did not miss any admissions or relevant variables.

### Statistical analyses

For each surveillance indicator, we plotted the absolute count per week during the study period and visually compared trends. Next, we plotted the incidence as a proportion of the total number of hospitalised patients for a specific week (incidence proportion). For the number of PCR tests, for example, we plotted the number of unique patients who were tested for at least one respiratory virus using an RT-PCR test, as the proportion of all newly hospitalised patients, for each week. A subanalysis including only patients that were admitted to the intensive care unit (ICU) at any point during their hospital admission was performed. In a second subanalysis, the results for RT-PCR tests and contact and droplet precautions were split by age group. We estimated the Pearson correlation coefficient between the different surveillance indicators over several time periods. Finally, we plotted the number of positive RT-PCR tests for each week in the study period. R software (version 4.3.1, R Foundation) was used to analyse the data and create the graphs.

## Results

A total of 417,119 hospitalisations were registered at Leiden University Medical Center between 1 January 2017 and 30 April 2023. Of these, 299,715 re-admissions and admissions with a duration of less than 24 h were excluded. A total of 117,404 admissions were included in our analysis. The flowchart of inclusion and exclusion can be found in Supplementary Figure S1. Information on data validation can be found in the Supplement. In our study period, 11,959 RT-PCR tests for respiratory viruses were registered, 4,683 contact and droplet precautions were registered, and 3,908 ICD-10 diagnostic codes of interest were registered. The overlap between the presence of the different surveillance indicators in the three different time periods can be seen in Supplementary Figure S2, S3 and S4. There were no missing data for any of the collected variables in our analysis.

### Absolute counts

**Figure 1:**
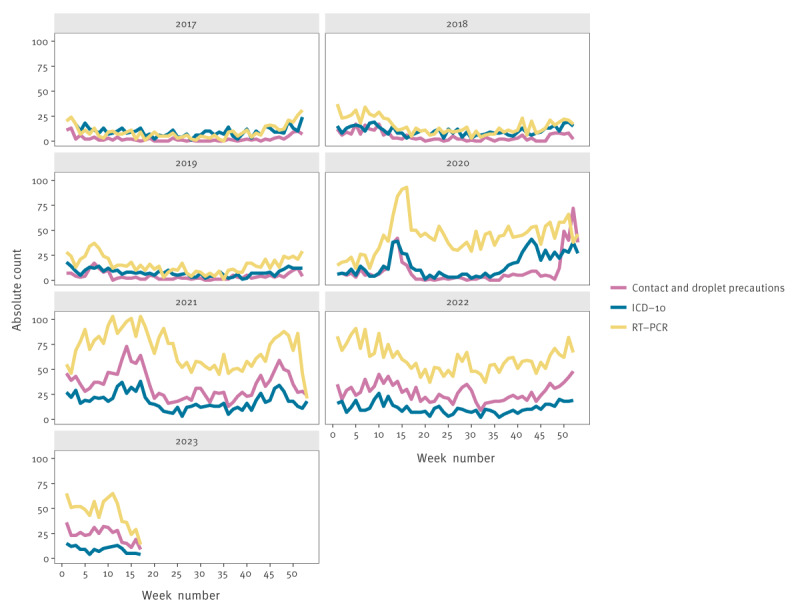
Absolute count per week for the three surveillance indicators over time, Leiden University Medical Center, Leiden, the Netherlands, 1 January 2017–30 April 2023 (n = 117,404 hospital admissions)

On average, the absolute count in the pre-COVID-19 years (2017–19) was lower than the absolute count during and at the end of the pandemic (see Figure 1 and Supplementary Table S1, which provides the mean count per week for the three surveillance indicators). Prior to the COVID-19 pandemic, all three surveillance indicators had relatively similar absolute counts, with the number of RT-PCR tests being slightly higher than the other two surveillance indicators, especially during the traditional influenza-like illness (ILI) season. In 2018, for example, there were on average 4.5 contact and droplet precaution labels, 10.2 ICD-10 codes and 15.1 RT PCR tests each week, see Supplementary Table S1. From spring 2020 onwards, the number of RT-PCR tests was consistently higher than the number of the other two surveillance indicators e.g. in 2021, there were 34.5 contact and droplet precaution labels, 18.2 ICD-10 codes and 69.6 RT-PCR tests each week (see Supplementary Table S1). The number of contact and droplet precaution labels remained low throughout most of 2020, but increased steeply at the end of 2020 and remained higher than ICD-10 registrations in 2021, 2022 and 2023 (up to and including week 18).

During this study period, 11,959 PCR tests were done, and 4,683 contact and droplet isolation labels and 3,908 ICD-10 codes were registered. The total number of registrations for the three surveillance indicators combined is 20,550 for 117,404 hospital admissions.

**Figure 2:**
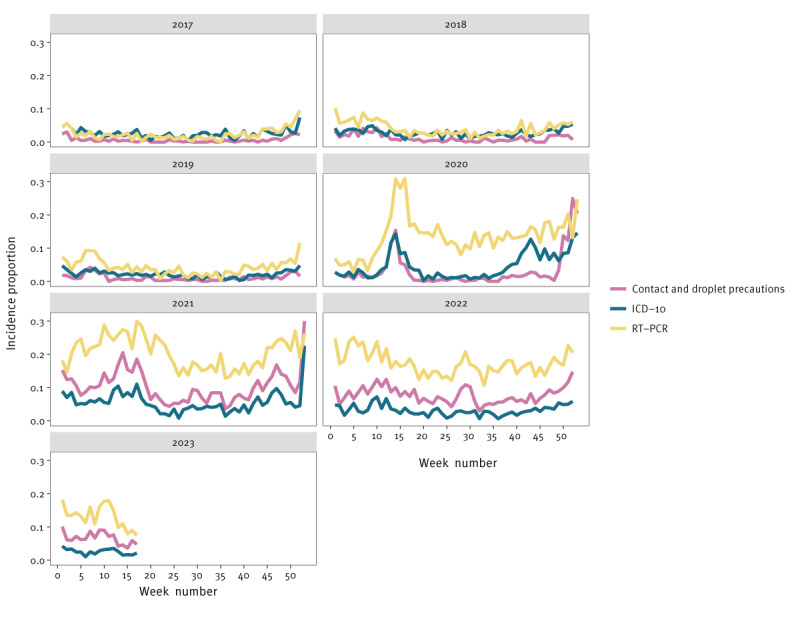
Weekly incidence proportion for the three surveillance indicators over time, Leiden University Medical Center, Leiden, the Netherlands, 1 January 2017–30 April 2023 (n = 117,404 hospital admissions)

When showing the incidence proportion, a similar pattern can be seen (Figure 2). The RT-PCR test incidence proportion noticeably diverges from the other two surveillance indicators after the first COVID-19 cases were reported in the Netherlands in week 9 2020 [13].

During this study period, 11,959 PCR tests were done, and 4,683 contact and droplet isolation labels and 3,908 ICD-10 codes were registered. The total number of registrations for the three surveillance indicators combined is 20,550 for 117,404 hospital admissions. Note that the y-axis ends at 0.3 instead of 1.0 to enhance visibility of differences between the different surveillance indicators.

### Virological test results

**Figure 3:**
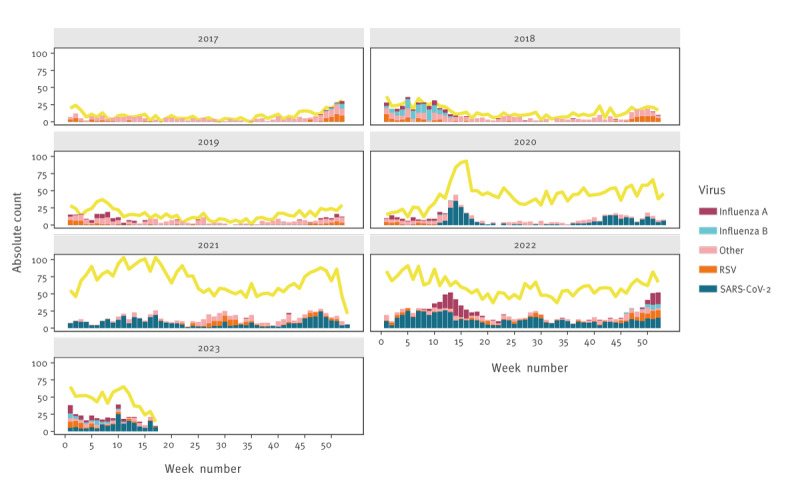
RT-PCR test results for respiratory viruses over time, Leiden University Medical Center, Leiden, the Netherlands, 1 January 2017–30 April 2023 (n = 117,404 hospital admissions)

RSV: respiratory syncytial virus; SARS-CoV-2: severe acute respiratory syndrome coronavirus 2.

Figure 3 shows the positive virological test results per week. Since the onset of the COVID-19 pandemic in 2020, there was an increase in the number of overall RT-PCR tests performed, accompanied by a lower proportion of positive test results. The number of RT-PCR tests that had an unknown test result, e.g. because the test was lost or the analysis was stopped, was stable over time, with roughly one unknown test result each week (data not shown). We therefore only show the positive test results over time, as a proportion of the total number of tests done.

Positive RT-PCR test results over time (coloured bars) and total number of RT-PCR tests done (yellow line) are shown. The total number of positive tests represented are as follows: influenza A: 274; influenza B: 114; other: 1,009; RSV: 317; SARS-CoV-2: 1,034. The ‘other’ group includes the following viruses: human adenovirus, bocavirus, human coronaviruses (229E, HKU1, NL63 and OC43), human metapneumovirus, parainfluenza virus 1–4 and human rhinovirus. The number of positive tests can be higher than the total number of tests when a patient tests positive for multiple viruses.

### Correlation

The correlation between the different surveillance indicators can be seen in the Table. The total number of RT-PCR results was used for the PCR surveillance indicator (as opposed to the number of positive tests). Overall, the correlation between the different surveillance indicators was highest in the third time period, especially between contact and droplet precautions and ICD-10 registration. 

**Table:** Pearson correlation coefficient for the correlation between combinations of surveillance indicators in the three different time periods, Leiden University Medical Center, Leiden, the Netherlands, 1 January 2017–30 April 2023

Surveillance indicators	Week 1/2017–week 8/2020	Week 9/2020–week 53/2020	Week 1/2021–week 18/2023
RT-PCR and contact and droplet precautions	0.82	0.38	0.73
Contact and droplet precautions and ICD-10	0.52	0.66	0.84
RT-PCR and ICD-10	0.57	0.56	0.64

The Pearson correlation coefficient reflects the correlation between two measures, with a value of −1 or 1 reflecting perfect positive or negative linear correlation, and a value of 0 indicating that there is no relation between the two measures.

### Intensive care unit population

A total of 4,847 ICU (including the neonatal ICU, NICU) admissions, with or without one of the three surveillance indicators, were registered in our study period. A sensitivity analysis including only ICU admitted patients was performed. In contrast to the total hospital population, the three different surveillance indicators followed a relatively similar trajectory over time in both the absolute number (provided in Supplementary Figure S5) and the surveillance indicators as a proportion of the number of ICU admissions (provided in Supplementary Figure S6).

### Result by age group

The three surveillance indicators show a relatively similar pattern when splitting the results by age group (provided in Supplementary Figures S7 (RT-PCR-tests), S8 (contact and droplet precautions) and S9 (ICD-10 diagnostic codes)). An increase in the number of RSV infections was reported in the summer of 2021 [14], which can be seen in the 0–4-year age group for all surveillance indicators, but most clearly with contact and droplet precautions (provided in Supplementary Figure S8) and ICD-10 registration (provided in Supplementary Figure S9). Using the positive RT-PCR test results provided in Supplementary Figure S10, the increase in RSV infections in young children can also be seen.

## Discussion

We compared three different surveillance indicators for their potential use in SARI surveillance. Generally, the three surveillance indicators followed a similar pattern between 2017 and 2023, with differences in the absolute values. There are two important exceptions to this, both in late 2020. The first was a divergence of the absolute count of contact and droplet precaution labels from the other two indicators, explained by a change in registration policy. This registration policy change involved an expansion of the number of healthcare workers who could register contact and droplet precautions. Prior to this change, only staff in the hospital hygiene and infection prevention department were able to register these labels. As registration of contact and droplet precaution labels is now primarily done by nurses, the registration is more complete as it is no longer limited to working hours, i.e. 9 am–5 pm Monday to Friday. The second exception is that during the second COVID-19 wave in the Netherlands (first surge started week 41/2020 [15]), there was an increase in ICD-10 registrations and positive RT-PCR tests, but not in the number of RT-PCR tests. The lack of increase in the number of RT-PCR tests might be explained by a relatively large number of patients who tested at home or were transferred to our hospital, in which case the test was not repeated. Moreover, using age-stratified analysis provides important additional information otherwise missed by the aggregated analysis, as was seen by RSV peak in young children in summer 2021.

Using a proxy for SARI surveillance, most commonly ICD-10 codes, is not new [5-7]. Germany has established a SARI surveillance system using ICD-10 diagnostic codes (J09–J22) [5]. Portugal [6] and Scotland [7] also used ICD-10 codes but include additional diagnostic codes to the list Germany used. Portugal for example, also included diagnostic codes for cardiovascular diseases (I20–I25, acute myocardial infarction; I50 and I51, heart failure), ILI symptoms (R05, cough; R51, headache; M79.1, myalgia; among others) and respiratory diagnosis or infection (I40.9, myocarditis; A49.9, bacterial infection; J45, asthma; and more). Scotland also included U07.1, U07.2 (COVID-19) and J04 (acute laryngitis and tracheitis). However, the use of the number of RT-PCR tests and contact and droplet precaution labels for surveillance is new and has specific advantages and disadvantages.

Firstly, RT-PCR tests for respiratory pathogens are widely used, especially in adults, and are likely to reflect that a patient presented with symptoms of a viral respiratory infection. A potential disadvantage of this indicator is that the number of RT-PCR tests is likely to be influenced by changing protocols. During the COVID-19 pandemic, the threshold for testing was low, and patients were frequently tested for the presence of respiratory viruses if they presented with fever, but no respiratory symptoms. In contrast, the low numbers year-round in the pre-pandemic years suggest that RT-PCR tests were only performed in specific patient groups. As protocols and public awareness could change again in the future, this can significantly influence the number of RT-PCR tests that are performed. Moreover, RT-PCR tests may not be performed if patients already tested at home, in another hospital or at their general practitioner. The large number of patients who only had a RT-PCR test, but no contact and droplet precaution labels or ICD-10 registration probably reflects a group of patients that had a negative RT-PCR test in the emergency department, where contact and droplet precautions were in place while waiting for the test result but not registered in the EMR.

Secondly, like RT-PCR tests, contact and droplet precautions are taken based on clinical presentation of a (viral) respiratory infection. The adherence to these precautions is likely to have improved since the start of the pandemic. A downside of using labels as a surveillance indicator is that they are also indicated in infections unrelated to SARI. These are mumps, pertussis, diphtheria, encephalitis, epiglottitis, parvovirus B19, meningitis, acute subglottic laryngitis, rubella and scarlet fever. However, compared with the numbers of patients with SARI, these numbers are extremely low. A second limitation of contact and droplet precaution labels is that registration may not always be correct in EMRs. This is less likely to happen with PCR testing as the request for testing is done within the EMR. However, this lack of registration is likely to be randomly distributed over time. There are several potential advantages of contact and droplet precaution labels over PCR as a surveillance indicator. The pressure on hospital capacity is an important aspect of surveillance, and is better reflected by contact and droplet precautions, as there is a direct relation between hospital capacity and the number of patients with contact and droplet precautions. In addition, in patients for whom a PCR test is considered too invasive, e.g. young children, contact and droplet precautions will still be in place.

A disadvantage that all surveillance indicators in our study have in common is that they are not, by definition, the same as a SARI diagnosis. Testing for respiratory viral infections have been done in asymptomatic patients, and an ICD-10 diagnosis of pneumonia does not guarantee that the patient had a fever and cough. Although we lose some precision with the surveillance indicators we selected, there are large advantages when it comes to feasibility: data collection can be done automatically using routinely collected data, with no additional administrative burden for healthcare staff. Many (Dutch) hospitals have EMRs that allow for automated extraction of data. The main goal of SARI surveillance is to monitor trends rather than absolute numbers [16]. While having precise numbers, e.g. not using a proxy, but identifying patients who meet the SARI definition, could be advantageous for identifying risk groups or assessing vaccine effectiveness, this would add considerable complexity to the surveillance system as more manual administration would be needed.

A possible explanation for the difference between the number of ICD-10 codes and the number of PCR tests and contact and droplet precaution labels is that the latter are done in patients with a suspected viral respiratory infection, while ICD-10 registrations are typically only used in confirmed infections.

While positive PCR test results provide important additional information, solely using this indicator skews surveillance towards pathogens that are most commonly tested for, potentially missing out on emerging or less common respiratory pathogens. Moreover, contact and droplet precautions – and the decision to test a patient using PCR – are based on clinical presentation, which better reflects the definition of SARI and incorporates clinical judgement. Both PCR testing and contact and droplet precaution labels could contribute to the detection of newly emerging pathogens. A large increase of either of these surveillance indicators without an increase in positive test results could indicate a new pathogen or variant (assuming similar testing and registration behaviour).

Our approach comes with several limitations. Firstly, as we do not have a gold standard for SARI in our dataset, it is not possible to indicate which surveillance indicator is most accurate. However, there is a strong correlation between the surveillance indicators, especially ICD-10 and contact and droplet precaution labels, and changes over time are still detectable. Comparing the proxies to the WHO case definition is an important future study objective, now that this proof-of-concept study demonstrated the potential use of these proxies for SARI surveillance. Secondly, data collection was performed retrospectively, while for a functioning, (near) real-time, surveillance system, data would be collected prospectively. For ICD-10 diagnostic codes especially, there is often a delay in registration, which could have a significant impact in a prospective setting. Thirdly, ICD-10 codes were only included if the same ICD-10 code was not registered within the previous year, to avoid the inclusion of patients with chronic disease. This could lead to a small underestimation of the number of SARI cases based on ICD-10 registrations. Finally, analysing data from multiple hospitals could confirm whether these indicators can be used for surveillance of SARI. As hospitals protocols may could change over time, regular validation of proxy indicators is essential. While the availability of contact and droplet precaution labels that can be extracted from EMRs may differ between countries and hospitals, our approach demonstrates that there are suitable proxies for SARI surveillance.

## Conclusion

The number contact and droplet precaution labels and ICD-10 codes are suitable for (automated) SARI surveillance in our cohort. PCR test results provide valuable additional information. Because of significant changes in public awareness and hospital testing policy, the number of RT-PCR tests is not considered a reliable indicator. As contact and droplet precautions best reflect pressure on hospital capacity, do not have a delay in reporting and registration policies are less likely to change over time, this parameter may be the most suitable indicator. Validating our results in different hospitals and in a prospective setting could confirm the feasibility of this indicator for SARI surveillance. An important future objective would be set up a national SARI surveillance system, collecting data from various hospitals across the country.

## Supplementary Data

480000 Supplement
