# Supplementary material for: Use of proxy indicators for automated surveillance of severe acute respiratory infection, the Netherlands, 2017 to 2023: a proof-of-concept study
Source: Euro Surveill. 2024 Jul 4;29(27):2300657. doi: 10.2807/1560-7917.ES.2024.29.27.2300657 (PMC11225262; doi:10.2807/1560-7917.ES.2024.29.27.2300657)
Supplement: Supplement [file 23-00657_SWETS_Supplement.pdf]

Supplementary information

This supplementary material is hosted by Eurosurveillance as supporting information alongside the article “Use of proxy indicators for automated surveillance of severe acute respiratory infection, the Netherlands, 2017 to 2023: a proof-of-concept study”, on behalf of the authors, who remain responsible for the accuracy and appropriateness of the content. The same standards for ethics, copyright, attributions and permissions as for the article apply. Supplements are not edited by Eurosurveillance and the journal is not responsible for the maintenance of any links or email addresses provided therein.

Results

Flowchart

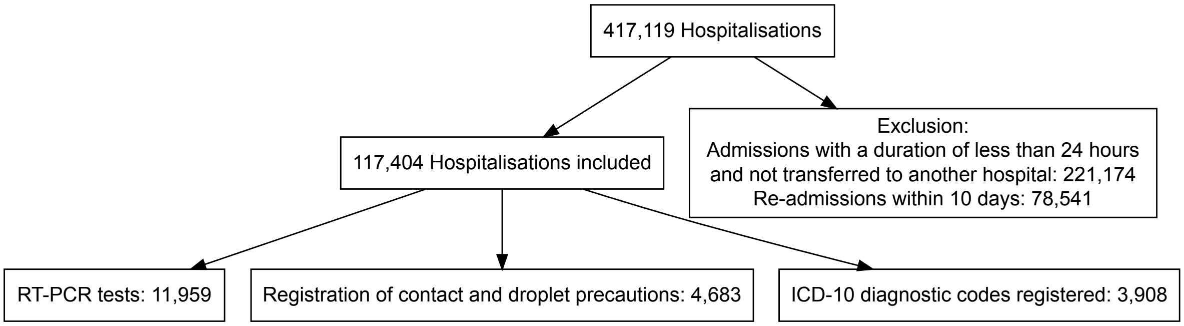

Supplementary Figure S 1: Flowchart of patient inclusion

Data validation

For each of the three indicator variables (PCR, Contact and droplet precautions and ICD-10 diagnostic codes) we selected two random weeks to compare our results to regular quality control data in our hospital. For all three surveillance indicators, we found the same patients in both our dataset and the quality control dataset.

Overlap between the different surveillance indicators

The overlap between the registration of contact and droplet precautions with the other two outcome measures has increased in the third timeperiod compared to the first and second timeperiod.

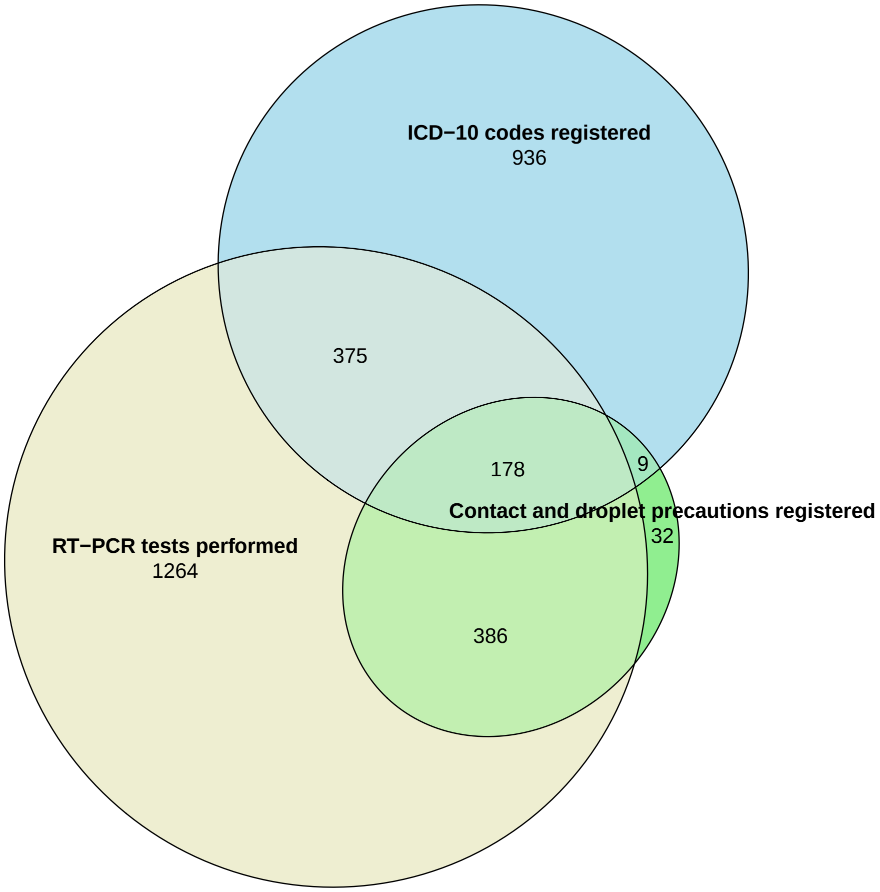

Supplementary Figure S 2: Overlap between the presence of the different surveillance indicators in the first timeperiod (week 1, 2017 - week 8, 2020).

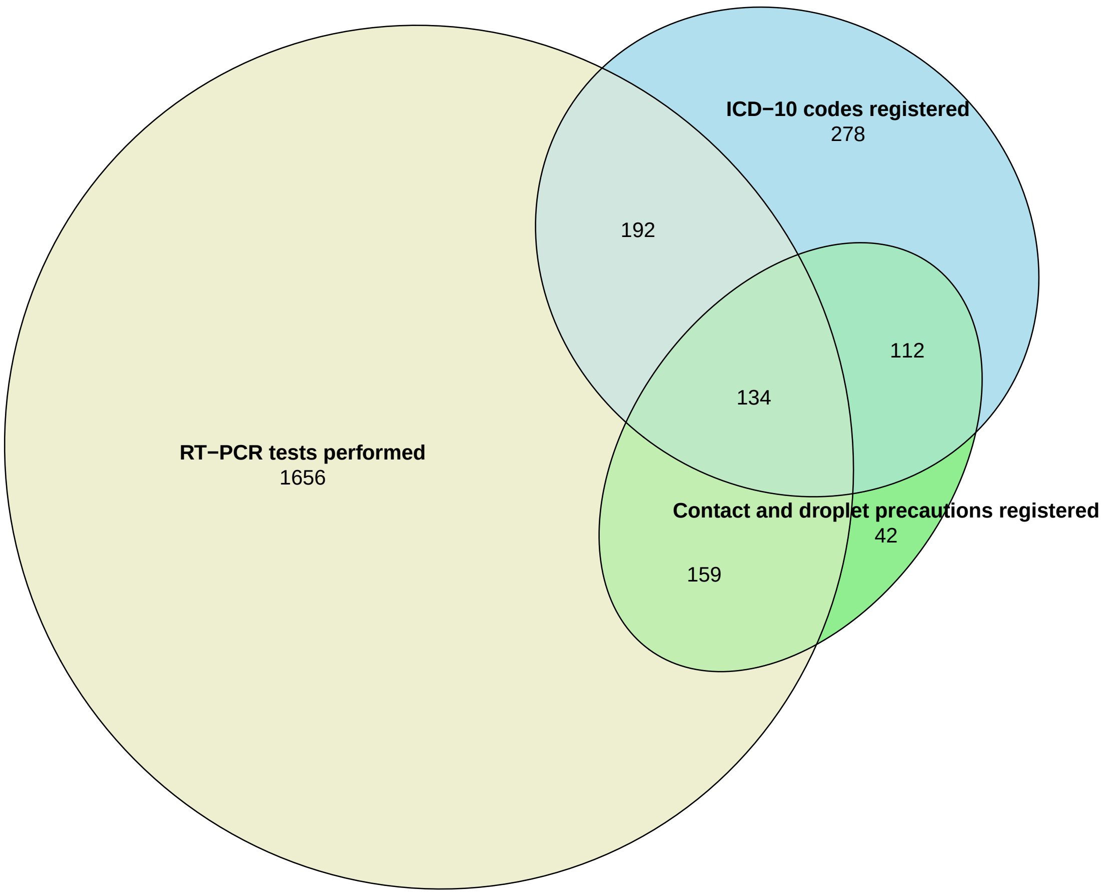

Supplementary Figure S 3: Overlap between the presence of the different surveillance indicators in the second timeperiod (week 9, 2020 - week 53, 2020).

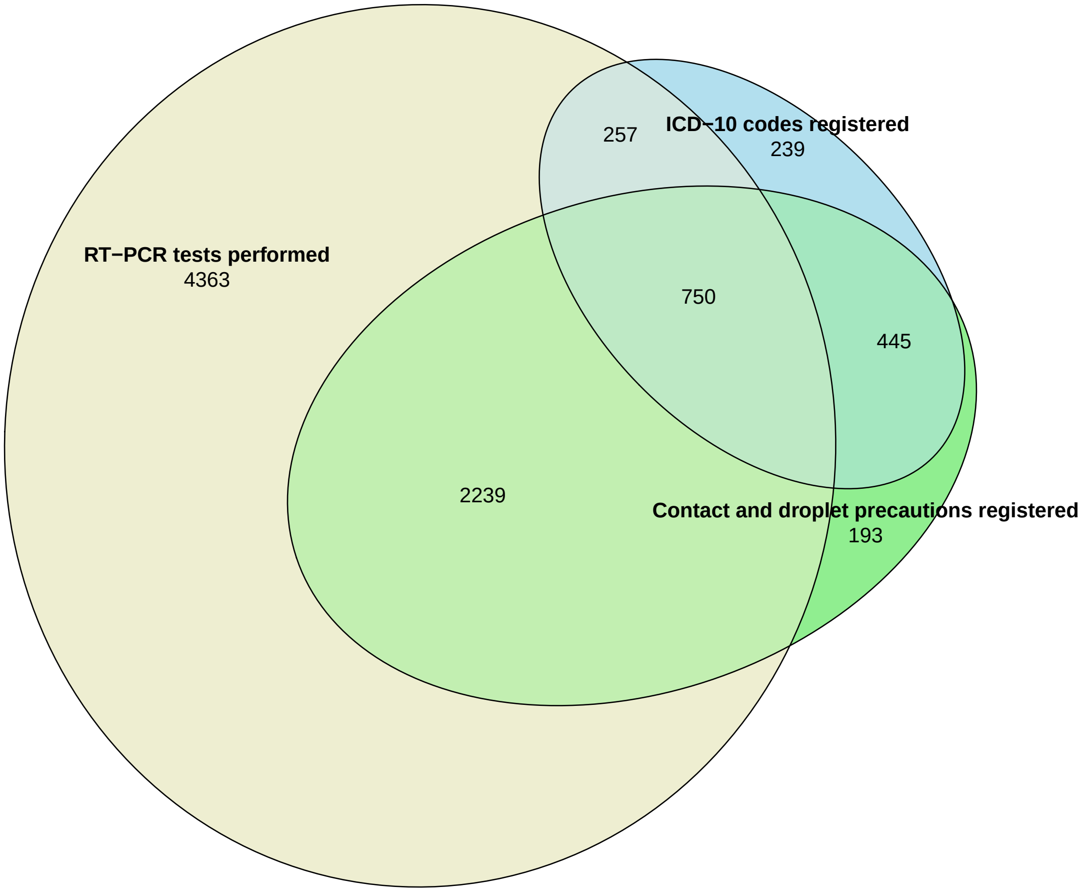

Supplementary Figure S 4: Overlap between the presence of the different surveillance indicators in the third timeperiod (week 1, 2021 - week 18, 2023).

Mean count per week

Year	Contact and droplet precautions	ICD-10	RT-PCR
2017	2.3	9.7	9.1
2018	4.5	10.2	15.1
2019	3.9	7.7	15.3
2020	9.4	14.7	43.2
2021	34.5	18.2	69.6
2022	26.9	11.0	60.2
2023	23.4	9.0	46.4

Year	Contact and droplet precautions	ICD-10	RT-PCR

Supplementary Table S 1: Mean number of registrations per week for each surveillance indicator, for each year of the study

Sensitivity analyses

ICU

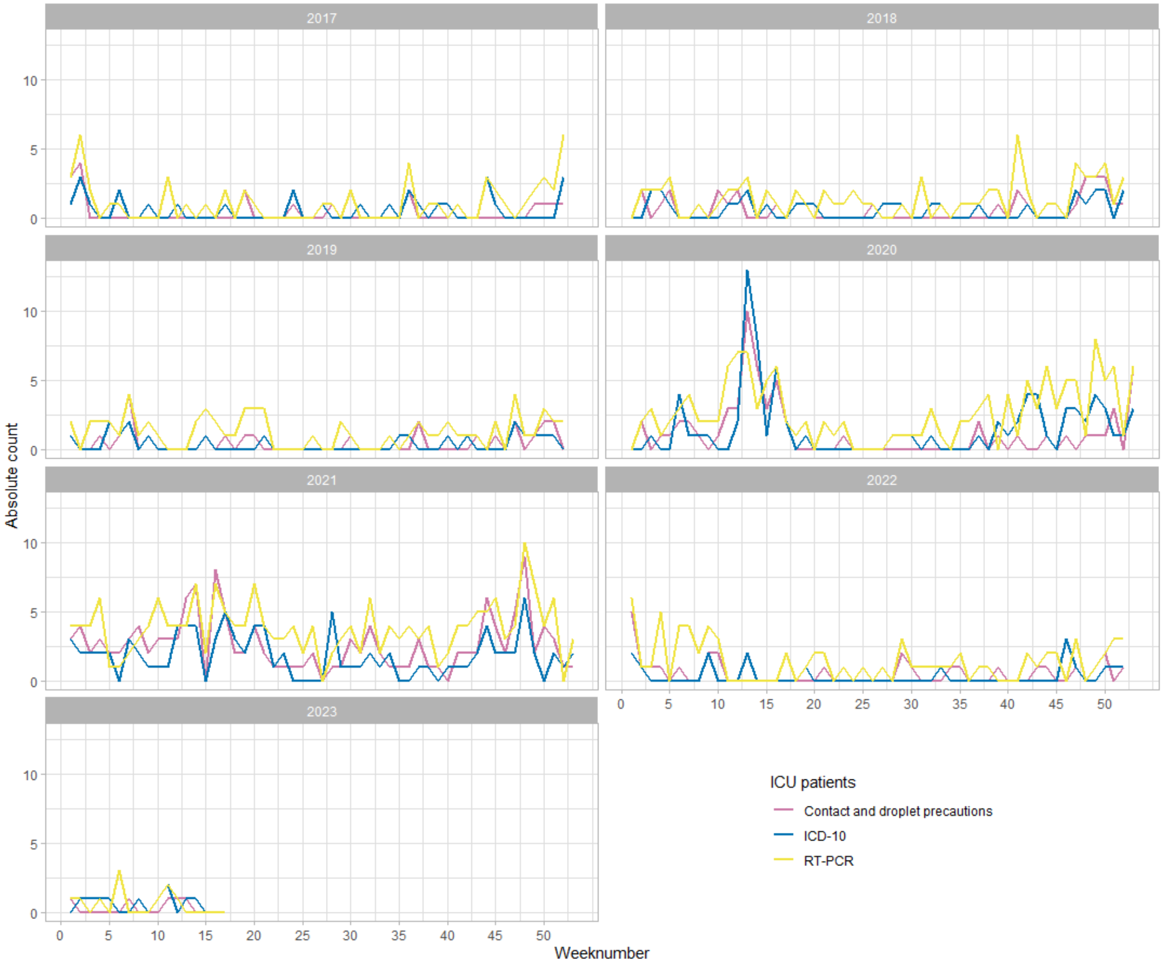

Supplementary Figure S 5: Absolute counts per week for the different surveillance indicators over time in ICU admitted patients.

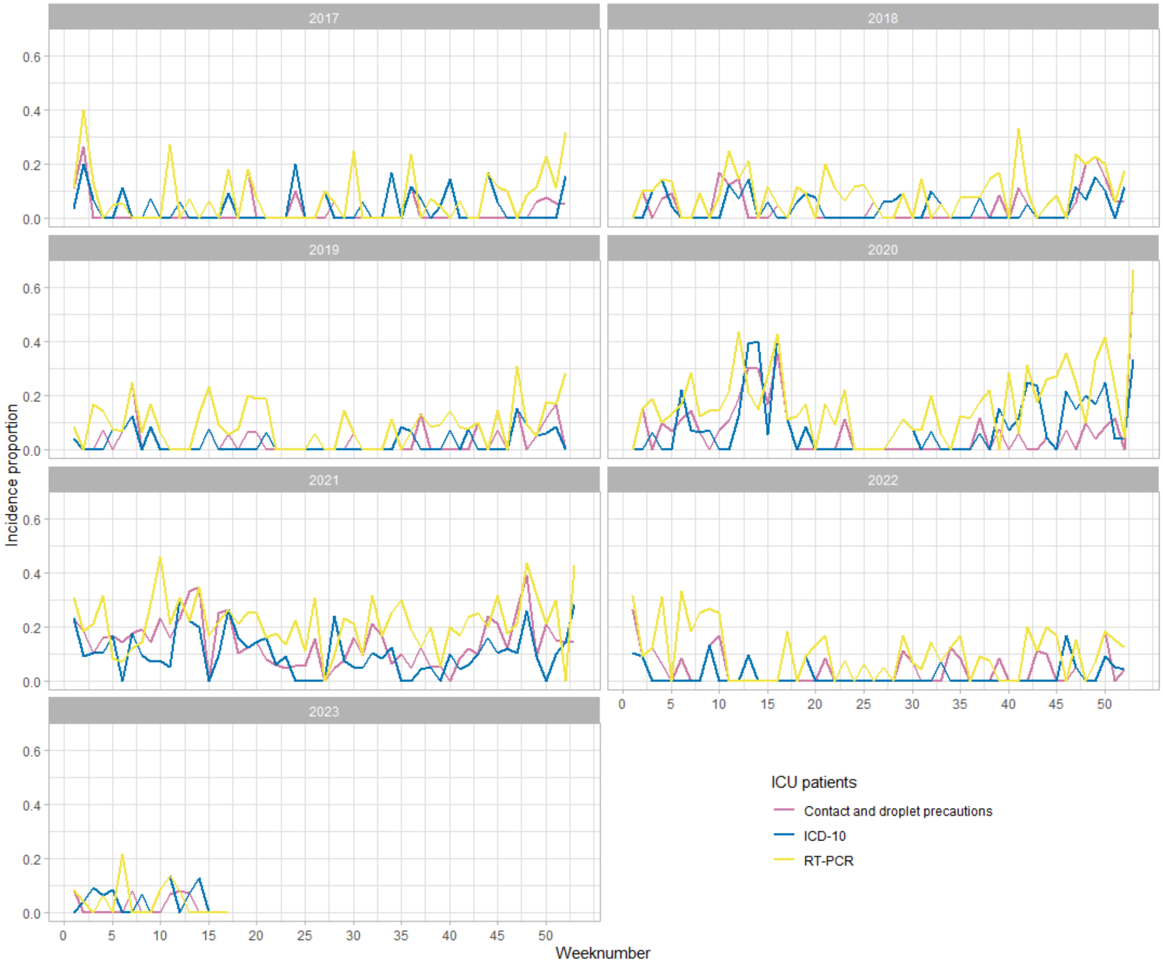

Supplementary Figure S 6: Weekly incidence proportion for the different surveillance indicators, in ICU admitted patients. Note that the Y-axis ends at 0.3 instead of 1.0 to enhance visibility of differences between the different survival indicators.

Age groups

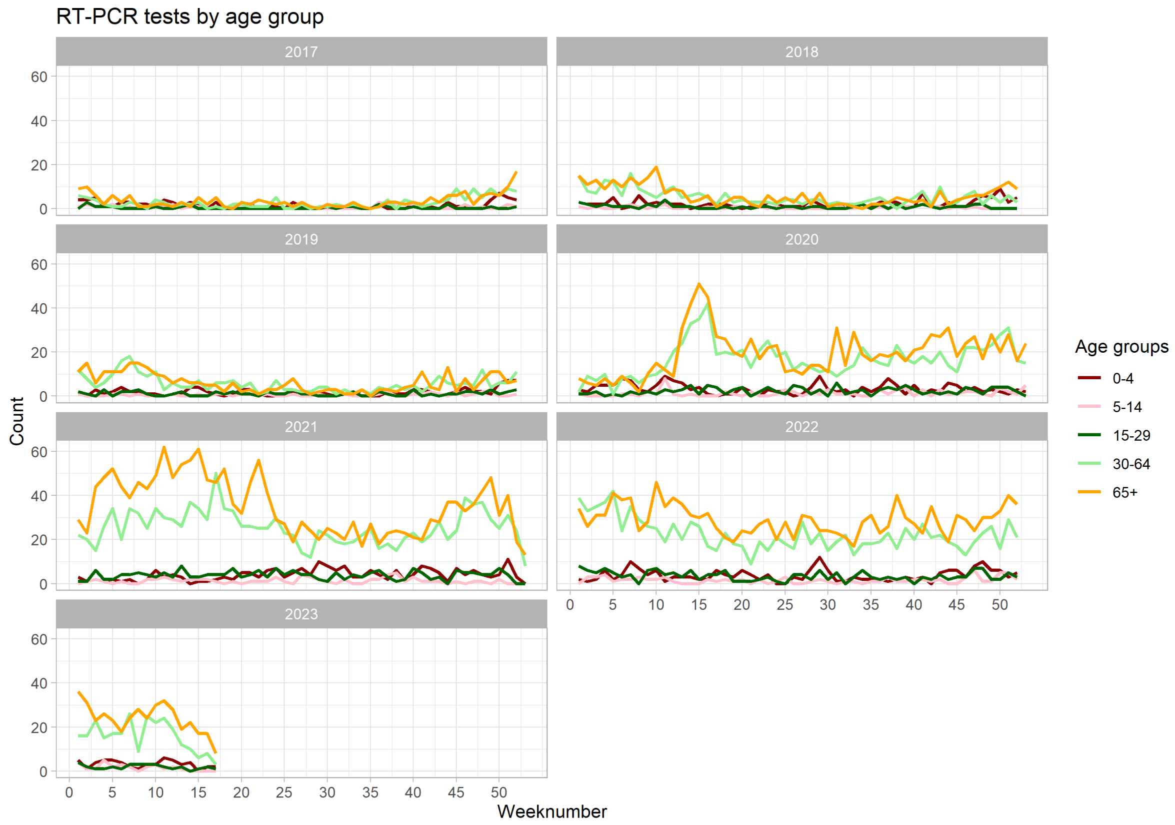

Supplementary Figure S 7: Weekly count of PCR tests, split by age group.

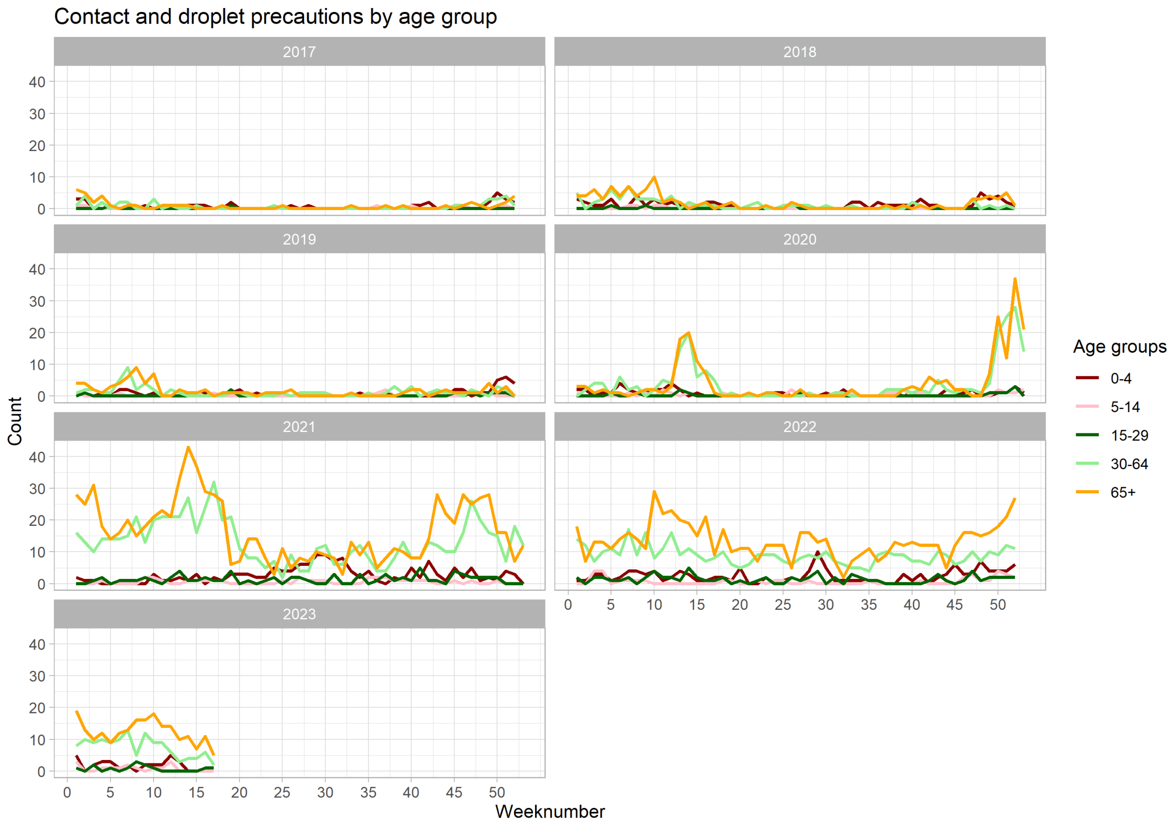

Supplementary Figure S 8: Weekly count of registration of contact and droplet precautions, split by age group.

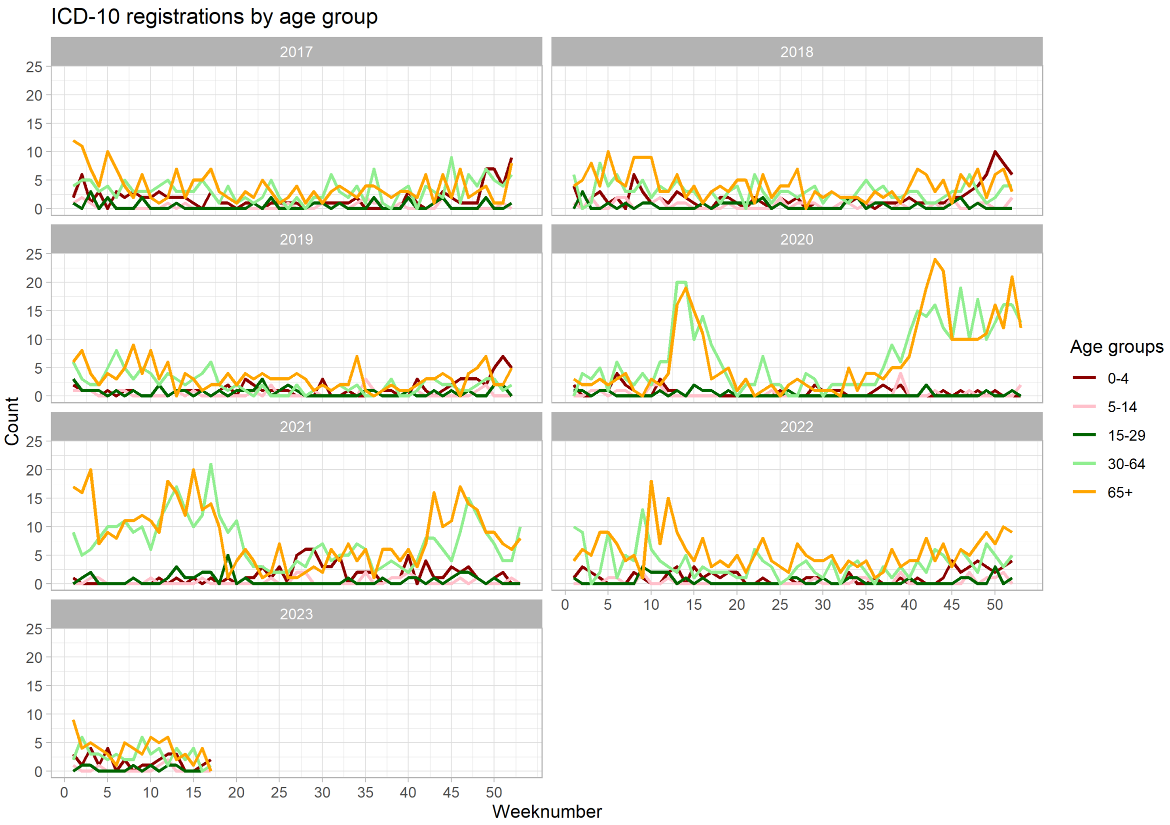

Supplementary Figure S 9: Weekly count of ICD-10 registrations, split by age group.

Positive RSV test results

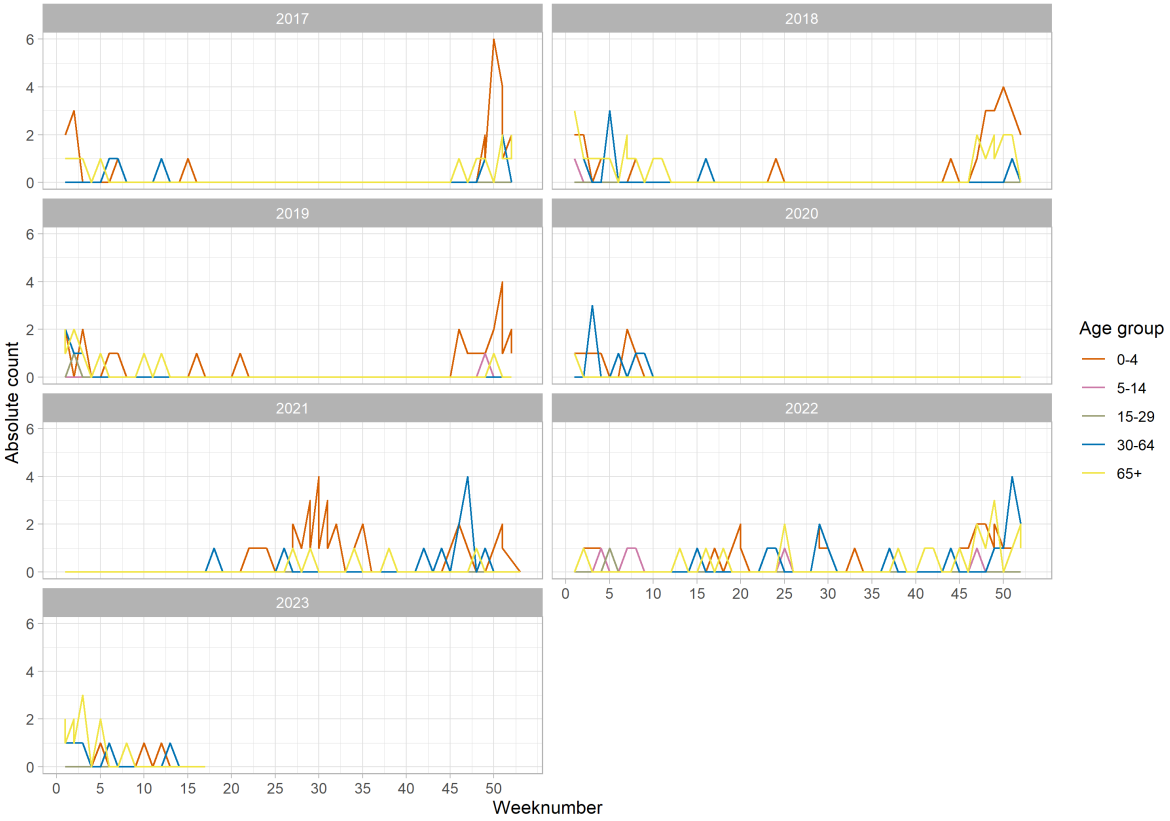

Supplementary Figure S 10: Positive RSV tests over time, split by age group.
